# Potential dosimetric error in the adaptive workflow of a 1.5 T MR-Linac from patient movement relative to immobilisation systems

**DOI:** 10.1007/s13246-023-01369-7

**Published:** 2024-01-16

**Authors:** Min Liu, Bin Tang, Lucia Clara Orlandini, Jie Li, Xianliang Wang, Qian Peng, David Thwaites

**Affiliations:** 1 Radiation Oncology Department, Sichuan Cancer Hospital & Institute, Affiliated Cancer Hospital of University of Electronic Science and Technology of China, Chengdu, China; 2 Radiation Oncology Key Laboratory of Sichuan Province, Sichuan Clinical Research Center for Cancer, Chengdu, China; 3 Institute of Nuclear Technology and Automation Engineering, Chengdu University of Technology, Chengdu, China; 4 Institute of Medical Physics, School of Physics, University of Sydney, Sydney, NSW Australia; 5 Sydney West Radiation Oncology Network, Crown Princess Mary Cancer Centre, Sydney, NSW Australia; 6 Radiotherapy Research Group, Leeds Institute of Medical Research, St James’s Hospital and University of Leeds, Leeds, UK

**Keywords:** Adaptive radiotherapy, MR-linac, Rectum cancer, Thermoplastic masks, Patient positioning systems

## Abstract

In magnetic resonance- (MR-) based adaptive workflows for an MR-linac, the treatment plan is optimized and recalculated online using the daily MR images. The Unity MR-linac is supplied with a patient positioning device (ppd) using pelvic and abdomen thermoplastic masks attached to a board with high-density components. This study highlights the dosimetric effect of using this in such workflows when there are relative patient-ppd displacements, as these are not visualized on MR imaging and the treatment planning system assumes the patient is fixed relative to the ppd. The online adapted plans of two example rectum cancer patients treated at a Unity MR-linac were perturbed by introducing relative patient-ppd displacements, and the effect was evaluated on plan dosimetry. Forty-eight perturbed clinical adapted plans were recalculated, based on online MR-based synthetic computed tomography, and compared with the original plans, using dose-volume histogram parameters and gamma analysis. The target volume covered by the prescribed dose (${\text{D}}_{\text{p}\text{r}\text{e}}$) and by at least 107% of ${\text{D}}_{\text{p}\text{r}\text{e}}$ varied up to − 1.87% and + 3.67%, respectively for 0.5 cm displacements, and to − 3.18% and + 4.96% for 2 cm displacements; whilst 2%–2 mm gamma analysis showed a median value of 92.9%. The use of a patient positioning system with high-density components in a Unity MR-based online adaptive treatment workflow can introduce unrecognized errors in plan dosimetry and it is recommended not to use such a device for such treatments, without modifying the device and the workflow, followed by careful clinical evaluation, or alternatively to use other immobilization methods.

## Introduction

Magnetic resonance guided adaptive radiation therapy (MRgART), using hybrid MR-linacs [1] modifies the RT treatment plan based directly on daily online MR imaging (MRI). This uses MRI’s inherently good soft tissue contrast and takes into account the actual patient anatomy for each treatment fraction [2]. MRgART is a promising methodology for many soft tissue cancer pathologies, including rectal cancer and is increasingly used for stereotactic body radiation therapy (SBRT) in such sites [3, 4]. Also, the advantages of using immobilization systems for the pelvic region treatments are widely recognized and recommended; often vacuum bags or equivalent knee and feet supports are used [5, 6], whilst in China thermoplastic masks [7, 8] are generally preferred.

Adapted plans based on daily MRI require the assignment of a relative electron density (ED) map for accurate dose calculation. The treatment planning system (TPS) provided as part of the Unity 1.5 Tesla MR-linac system (Elekta, Crawley, UK) is the Monaco TPS (Elekta AB, Stockholm, Sweden). When calculating MR-based synthetic computed tomography (sCT) plans for patients treated on a Unity MR-linac, the sCT strategy applied in Monaco is to use bulk density assignment based on the contours from the original patient simulation CT. This includes the contours of the different components of the patient positioning device (ppd). During the online adapt-to-shape (ATS) procedure, the daily acquired MRI is registered to the simulation CT or MRI from a previous session and the contour information, including average EDs and their layering (prioritisation), is propagated to MRI to generate a sCT by ED assignment on the MRI. The adapted plan is reoptimized and recalculated on the sCT, so the dosimetric accuracy of this strategy needs accurate evaluation [1, 9–11].

The dosimetric effects of any ppd must be investigated carefully for use in such a complex workflow, especially if it has high-density components that can impinge on the treatment field. Furthermore, ppds as generally supplied cannot be visualised on MR images and therefore may not be accurately included in MR-only clinical adaptive workflows. In particular, a ppd used to lock a thermoplastic mask may present high-density components, but the patient is not rigidly connected to the mask and support and is not completely stationary relative to the ppd plate. The patient is constrained by their individually modelled mask, which is fixed to the ppd, but despite care in this procedure, small patient shifts within the mask are still possible and are not visible on MRI. In addition, the ppd plate should be represented as a rigidly attached part of the Unity couch. However, the latest versions of the Monaco TPS still consider it as a structure lying on top, assuming the patient stays in the same position relative to it.

Shifts may be a particular concern when considering the long times for online MRgART sessions [1, 2, 12], where the patient has to remain still on the treatment couch for mean times of at least 45 min and up to 60 min [3, 13]. Intrafraction setup shifts have been widely investigated for different types of immobilisation systems, including thermoplastic masks [14–17] and including for pelvic treatment [18, 19]. However, these are mostly for shorter sessions than in MRgART.

This study aims to highlight to the community the potential dosimetric effect of such limitations in the Unity MR-linac system (including Monaco TPS) adapted treatment workflow, when using a ppd with high-density components. For this, relative shifts between patient and ppd were simulated and the dosimetry of the perturbed and reference-adapted plans was compared.

## Materials and methods

Our Unity 1.5T MR-linac system includes Monaco v5.4 and was supplied together with a ppd based on the Klarity R612-MR multifunctional fixing frame (Klarity Medical & Equipment Co., Guangzhou, China) for x thermoplastic pelvic masks. The first two rectum cancer patients (pt1 and pt2) treated on this system and set up with pelvic thermoplastic masks were included in this retrospective study. Pre-clinical use tests of the ppd, on a homogeneous phantom, showed correct delineation of the ppd and its components and accurate bulk density assignment; comparison of optimized plans on the reference CT scan and recalculated (not reoptimized) plans on the sCT generated by the bulk density assignment showed greater than 99.5% agreement on gamma analysis with 2%–2 mm criteria. Nevertheless, during the first MR-based online adaptive sessions it was recognised that relative movements between the patient and the ppd were not fully visualised or considered in a realignment of the systems, as they would be for x-ray based image-guided systems. All procedures and methods were carried out in accordance with relevant guidelines and regulations; informed consent was obtained from all subjects. The study was approved by the institutional Ethics Committee (approval number: SCCHEC-02-2022-003, January 4, 2022).

### Use of ppd for thermoplastic masks

**Fig. 1:**
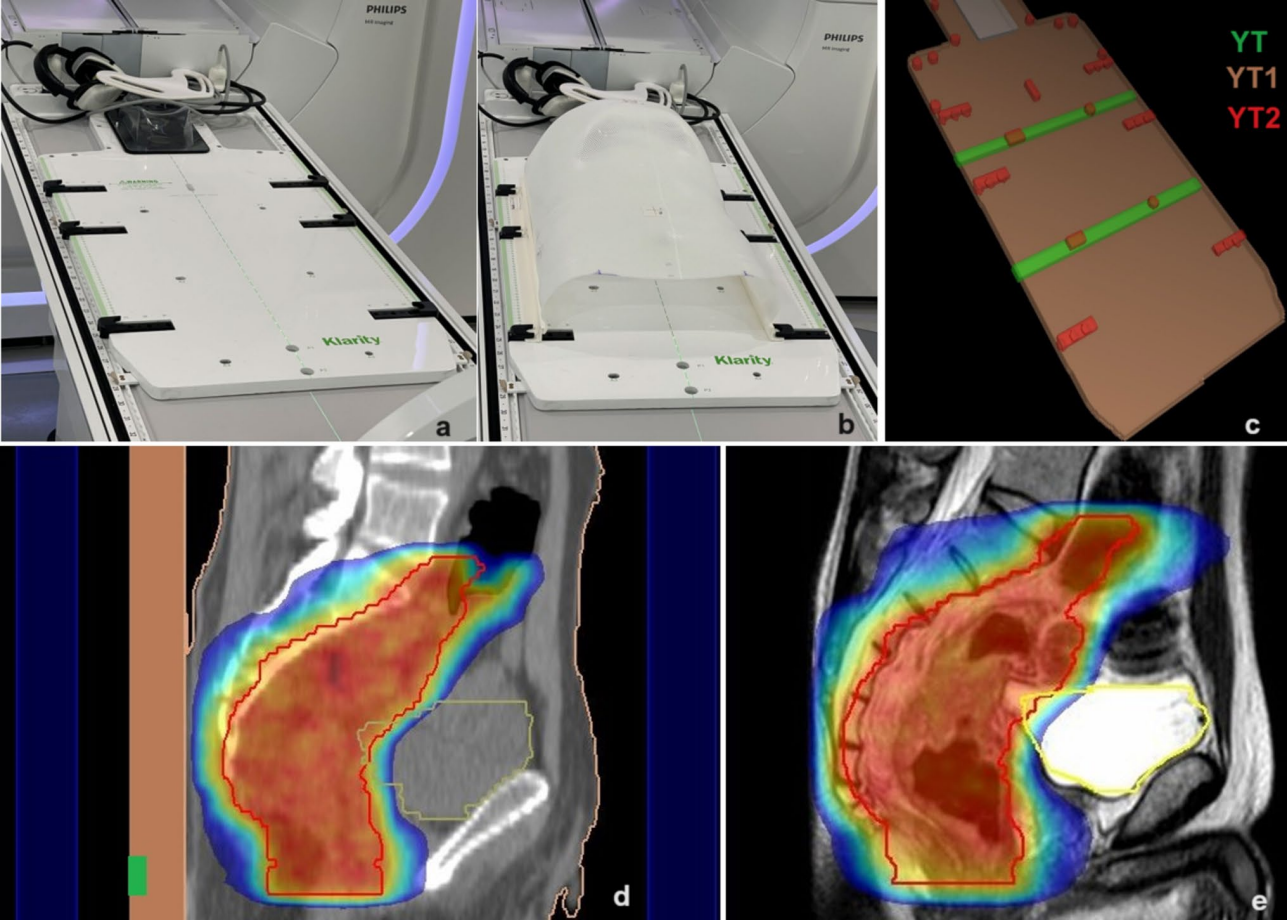
Patient positioning system **a**, **b** and its components YT, YT1, and YT2 in a 3D view **c**. ppd visibility **d** and non-visibility **e** on representative sagittal CT, and MRI images, respectively

The Klarity ppd plate (Fig. 1) was used to fix the thermoplastic pelvic masks. It comprises three distinct parts, YT, YT1, and YT2 with electron densities 0.476, 0.360, and 0.807 g/cm^3^, respectively, as evaluated by the Monaco TPS. YT1 is the main plate, inside which there are two bars at the top and bottom (YT) and additional smaller parts (YT2) that fix the plate to both the couch and the mask. In clinical practice the ppd is rigidly attached to the couch at the same indexed bar position; however, in Monaco TPS 5.4 used for this study and in the latest version 5.40.04 released for Unity, the ppd is not considered part of the couch or rigidly attached to it. In the TPS, the ppd and the external contour of the patient can be combined to form an outline, or the ppd can be considered as a patient structure. During the online adaptive procedure, before proceeding to the MR-based sCT calculation, the contours are adjusted by the radiation oncologist to the anatomy seen on the MRI, but relative patient-ppd shifts cannot be managed because the ppd is not visible on the MRI. A change in the relative position of the target or organs at risk (OARs) relative to the high-density parts of the ppd may lead to inappropriate dosimetry. Figure 1 shows the ppd and its visibility/non-visibility on representative sagittal CT and MRI images.

### Patient workflow

Patients were set up in the supine position using indexed positioning aids. For pt1 and pt2, pelvic thermoplastic masks locked to the ppd were used. Once the problem reported here was recognised, subsequent rectum cancer patients were immobilised without masks to avoid the issue, using Wing-STEP, KneeSTEP, and FeetSTEP supports (IT-V, Innsbruck, Austria) instead. A bladder catheter was used at simulation and at each treatment fraction to ensure consistent filling [11, 12]. Simulation imaging was acquired on a Brilliance Big Bore CT (Philips Medical System, Cleveland, OH, USA) and the MR-linac’s scanner, consecutively on the same day, with the same immobilization devices. CT and MR imaging datasets were exported to Monaco for target and OARs delineation.

Target and OARs contouring followed UK SABR consortium guidelines [20]. The previously-delineated ppd contour was imported from the contours database and positioned to match the one displayed on the patient CT images. Patient prescription dose (${\text{D}}_{\text{p}\text{r}\text{e}}$) was 25 Gy in 5 fractions. The CT reference treatment plans were on the CT-imaging dataset to achieve the ${\text{D}}_{\text{p}\text{r}\text{e}}$ to 95% of the target volume keeping the OARs doses as low as possible [21, 22]. The plans were optimised using 10 individual beam angles (190°; 210°; 228°; 300°; 330°; 30°; 60°; 130°; 150°; 170°), a 3 mm dose grid and a 1% uncertainty per calculation. The CT reference plan includes all the information needed to generate the sCT during the online MR-based adaptive workflow, including average EDs and a given layering priority for each contour and ppd component. The accuracy of the calculation using the sCT generated by bulk density assignment is assessed for each patient within the clinical workflow [11].

Each treatment fraction starts by acquiring a first online MRI to use for plan adaptation. The pre-treatment CT (or previous session’s MRI scan), contours, plan, and the daily online MRI, are used as input to adapt the plan for that specific session. The MRI or CT scan used as reference is matched with the online MRI by rigid registration, and the reference data isocentre position is updated; then the workflow for rectum cancer patients uses an ATS approach based on the new patient anatomy and the adapted plan is recalculated on the daily MR-based sCT [11, 23]. A second MRI is acquired while approving the adapted plan and rigidly registered with the first to ensure the appropriateness of the ongoing treatment delivery, and a further MRI is acquired in real-time during the delivery [12].

### Evaluation of the ppd dosimetric impact

**Fig. 2:**
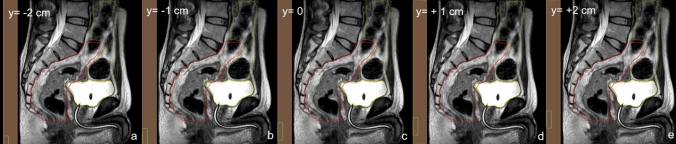
Simulated shifts of the ppd on the MRI acquired at the 2nd fraction for pt2. The ppd components YT1 (in brown) and YT (green rectangle) are shifted from the baseline position **c**, in the y direction by + 1 cm **d**, + 2 cm **e**, − 1 cm **b** and − 2 cm **a**. The target is in red, while the bladder and intestine are in yellow and light grey, respectively

For each of the two rectum patients treated using the thermoplastic masks, the adapted treatment plan (TP_ADT_) for each of the clinically delivered fractions originating from the daily MR-based sCT calculation, was recalculated (not reoptimised) without changing the MU or any other parameters, using a 2 mm dose grid and shifting the ppd in the longitudinal (y) direction by ± 0.5 cm, ± 1 cm, and ± 2 cm. The applied shifts are within the range potentially expected for setup uncertainties for non-cranial tumors, from a wide review of accuracy and uncertainty by van Dyk et al. [24]. They also reflect institutional reports of clinical experience of mean translational setup variations in the longitudinal direction for pelvic sites, ranging from less than 5 mm [25, 26], between 5 and 10 mm [23, 27] and more than 10 mm [27–29]. Figure 2 shows the MRI acquired at the second fraction for pt2, with introduced ppd displacements from its original position in the upper and caudal longitudinal direction.

The sCT was generated, assigning to each contoured region (including the ppd and its components) the average ED of the corresponding contours as on the reference CT before treatment planning. Target and OARs DVH dosimetric differences were assessed and compared with the corresponding unperturbed plan; particularly the volume receiving the prescribed dose (${\text{V}}_{\text{D}\text{p}\text{r}\text{e}}$), and the volume receiving at least 107% of the prescribed dose (${\text{V}}_{107{\%}}$) for the target, while for the OARs ${\text{t}\text{h}\text{e} \text{m}\text{a}\text{x}\text{i}\text{m}\text{u}\text{m} \text{d}\text{o}\text{s}\text{e} \left({\text{D}}_{\text{m}\text{a}\text{x}}\right), \text{a}\text{n}\text{d} \text{D}}_{0.5\text{c}\text{c}}$, ${\text{D}}_{5\text{c}\text{c}}$, ${\text{D}}_{10\text{c}\text{c}}$, i.e. the dose received by 0.5, 5.0, and 10.0 cm^3^ of the OARs volume, respectively, were considered. The percentage point changes used to compare target dosimetry is obtained by subtracting the values of the dosimetric parameter of the perturbed plan from the corresponding values of the reference adapted plan. Negative or positive percentage points correspond to a decrease or increase, respectively in the target volume covered by the indicated dose; whilst for the OARs, the absolute dose difference was considered.

Differences in dose distribution from the zero-displacement plan were assessed with global gamma analysis. DICOM-RT files including MR images, RT plans, RT structures and RT dose were exported to Matlab R2013a. Target and OARs dose distributions were assessed using gamma analysis with 2%–2 mm criteria and lower dose threshold of 5% of the maximum dose, using CERR v4.4 (https://github.com/cerr/CERR).

Following internal guidelines to keep the overall dosimetric discrepancy of the treatment as low as possible, the sCT treatment plan recalculated from the reference CT plan is considered in agreement when the target dose difference at any point of the DVH is lower than 1.5% or 1 Gy, as also reported in a previous study from our group [11]. Therefore, the potential DVH inconsistency due to a single contributing factor, such as the variation of the relative patient-ppd position investigated here, is expected to be below these values.

## Results

Pt1 completed the 5-fraction short course RT regimen and the adapted reference plans were optimized with total MU/segments of 1960/110, 2048/109, 2007/108, 2154/109, and 1942/110, respectively. Pt2 underwent three online adaptation sessions before the error introduced by the use of ppd became clear and the patient was moved to another linac; the MU/segments for each of the adapted reference plans were 1948/117, 2178/116, and 2235/120, respectively. A total of 30 and 18 perturbed adapted plans were recalculated for pt1 and pt2, respectively, using the MR-linac images acquired at each fraction and shifting the relative patient-ppd position.

**Fig. 3:**
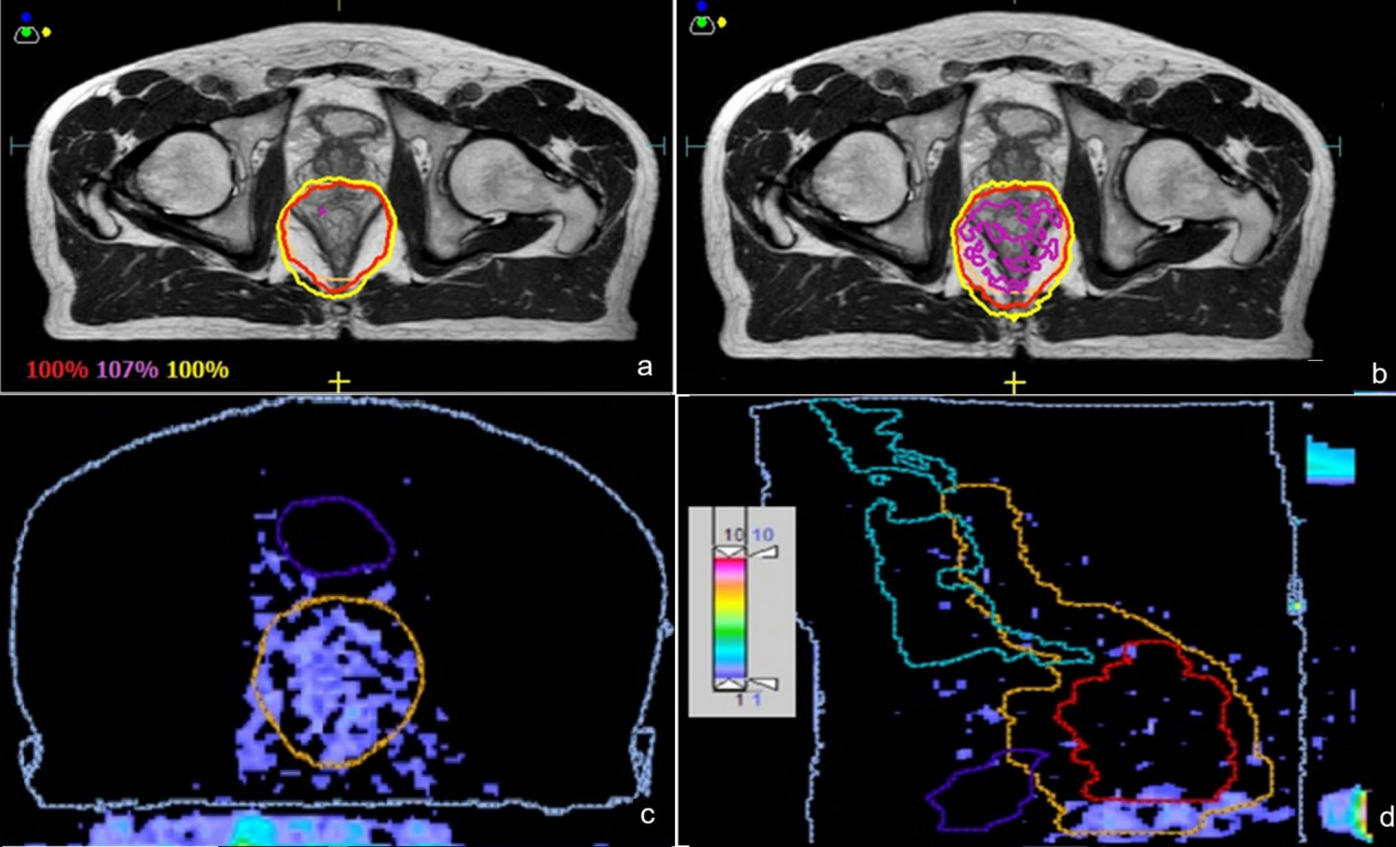
Comparison of the isodoses of the ADT3 delivered **a** and perturbed **b** adapted plans for pt1 on the transversal view, and corresponding gamma analysis in the transverse **c**, and sagittal **d** views of the caudal part of the target

For each fraction and adapted plan, the induced ppd-to-patient displacement impacted the dosimetry of the target as shown in Table 1. The difference between original and perturbed adapted plan target dosimetry for each shift produces a discrepancy in one of the dosimetric parameters, i.e. a reduction in the volume covered by the prescription dose, and/or an increase in target volume covered by 107% or more of the dose prescribed. The impact of relative YT-patient displacement on plan dosimetry is influenced not only by the daily reference position of the YT bar, but also by the concurrence of other factors, including the distance between the edge of the target and the YT bar over the different target slices and the recontoured target at each adjusted fraction. A shift from the reference position of the ppd towards the target from 0.5 cm up to 2 cm has the effect of decreasing the target coverage (${\text{V}}_{\text{D}\text{p}\text{r}\text{e}}$) by up to 1.87% and 3.18%, respectively, with average values (over all fractions considered) of 1.31% and 2.12%, respectively. Similarly, a shift in the opposite direction increases the magnitude of the target hot areas (${\text{V}}_{107\text{\%}}$) by up to 3.67% and 4.96% respectively, with average values of 1.70%, and 2.64%, respectively. Hot spots may appear when the adapted reference plan has been optimized with the YT bar at its reference position covering even partially the caudal part of the target. If the YT bar is moved away, the target area initially “obscured” by the bar will receive a higher dose. If this area was already close to 107% of the ${\text{D}}_{\text{p}\text{r}\text{e}}$, the dose may increase beyond 110% of the ${\text{D}}_{\text{p}\text{r}\text{e}}$. This is the case for the ADT3 fraction for a − 2 cm shift, where differences in isodoses are observed at or near the high-density parts of the ppd, with non-negligible overdoses as shown in Fig. 3.

The differences in the 2%-2 mm gamma analysis between the reference target dose distribution (y = 0) and the perturbed adapted plans are shown in Table 2; the gamma distribution ranged between 85.7% and 95.0% for pt1 and between 92.1% and 97.6% for pt2;

For OARs the dosimetric differences in the bladder (${\text{D}}_{0.5\text{c}\text{c}}$, ${\text{D}}_{10\text{c}\text{c}}$), small bowel (${\text{D}}_{0.5\text{c}\text{c}}$, ${\text{D}}_{5\text{c}\text{c}}$), colon (${\text{D}}_{0.5\text{c}\text{c}}$); femoral heads (${\text{D}}_{\text{m}\text{a}\text{x}}$, ${\text{D}}_{10\text{c}\text{c}}$), and the spinal cord (${\text{D}}_{\text{m}\text{a}\text{x}}$), showed a median value of 0.07 Gy (range, 0.0–0.81 Gy), where ${\text{D}}_{\text{x}\text{c}\text{c}}$ is the dose received by x cm^3^ of the volume. The gamma analysis (2%–2 mm) of the mentioned OARS showed median values of 98.74%.

**Table 1:** Reference vs. perturbed adapted plans, target dosimetric parameters percentage points change *in bold and percentage points decreased and increased, respectively by more than 1.5%

		Pt1-ADT1		Pt1-ADT2		Pt1-ADT3		Pt1 -ADT4		Pt1-ADT5		Pt2-ADT1		Pt2-ADT2		Pt2-ADT3	
	Δy(cm)	${\text{V}}_{\text{D}\text{p}\text{r}\text{e}}$	${\text{V}}_{107\text{\%}}$	${\text{V}}_{\text{D}\text{p}\text{r}\text{e}}$	${\text{V}}_{107\text{\%}}$	${\text{V}}_{\text{D}\text{p}\text{r}\text{e}}$	${\text{V}}_{107\text{\%}}$	${\text{V}}_{\text{D}\text{p}\text{r}\text{e}}$	${\text{V}}_{107\text{\%}}$	${\text{V}}_{\text{D}\text{p}\text{r}\text{e}}$	${\text{V}}_{107\text{\%}}$	${\text{V}}_{\text{D}\text{p}\text{r}\text{e}}$	${\text{V}}_{107\text{\%}}$	${\text{V}}_{\text{D}\text{p}\text{r}\text{e}}$	${\text{V}}_{107\text{\%}}$	${\text{V}}_{\text{D}\text{p}\text{r}\text{e}}$	${\text{V}}_{107\text{\%}}$
Reference	0	95.32	0.64	95.57	0.30	95.40	0.31	95.86	0.27	95.03	0.92	94.99	0.00	95.01	0.10	95.00	0.67
% Point Change	+ 2	**− 2.53**	0.05	− 0.95	**1.58**	**− 1.60**	**2.64**	**− 3.18**	0.34	**− 2.71**	0.26	**− 2.20**	0.20	**− 1.92**	0.17	**− 1.88**	**1.91**
	+ 1	**− 1.87**	0.01	− 0.70	**1.64**	-0.80	**2.67**	**− 1.51**	0.44	**− 1.66**	0.49	**– 1.59**	0.38	**− 1.47**	0.15	**− 1.87**	0.44
	+ 0.5	**− 1.87**	0.70	− 0.72	**1.62**	0.10	**3.00**	**− 1.50**	0.40	**− 1.70**	0.45	**− 1.58**	0.38	− 1.30	0.15	**− 1.86**	0.35
	-0.5	0.16	0.67	0.48	**1.49**	0.80	**3.67**	1.25	**1.90**	0.01	1.50	− 0.30	**2.25**	− 0.37	1.10	0.10	1.00
	-1	0.08	1.13	0.54	**3.02**	0.89	**3.78**	1.09	**1.91**	0.25	**1.54**	− 0.39	**2.20**	− 0.34	1.07	− 0.38	1.08
	-2	0.56	1.32	0.61	**3.47**	1.01	**4.96**	0.41	**2.66**	0.07	**1.77**	− 0.31	**2.38**	− 0.06	**2.11**	0.05	**2.46**

**Table 2:** Reference vs. perturbed adapted plans, target gamma analysis *in bold values lower than 95%. ADTi : adapted plan at fraction i

Shifts	ADT1		ADT2		ADT3		ADT4	ADT5
(cm)	Pt1	Pt2	Pt1	Pt2	Pt1	Pt2	Pt1	Pt1
+ 2	**87.7**	**93.7**	**94.0**	**93.0**	**93.8**	**92.1**	**85.7**	**91.9**
+ 1	**88.1**	**94.7**	**95.0**	**93.2**	**92.8**	**93.5**	**88.7**	**92.7**
-1	**92.2**	**94.5**	**90.6**	**94.7**	**89.4**	97.4	**92.9**	**92.7**
-2	**91.0**	**94.0**	**91.3**	**94.1**	**91.2**	97.6	95.0	**93.0**

## Discussion

MRgART on the Unity linac allows to recalculate and re-optimize the plan using the sCT generated from the daily MRI. It is crucial to put in place all possible actions to ensure the calculation accuracy on this MR-based sCT [1]. The Klarity ppd supplied with the Unity MR-linac system is intended to be used as an integral part of the workflow to achieve treatment reproducibility. Potential shifts of the patient within the thermoplastic mask, relative to the ppd cannot be observed in the current standard online adaptive workflow, as the ppd is not visible on MRI. In addition, the latest versions of the Unity system’s TPS (Monaco) still treat the ppd as an overlying structure not fixed to the couch and assume the patient is in the same position relative to it. Thus for adaptive sCT calculation using the daily online MRI, potential relative shifts between the patient/target/OARs and the ppd high-density components need to be taken into account in ways that aren’t enabled by the system as supplied.

There is a significant research literature assessing and evaluating positioning/shift errors in a range of treatment sites and with different immobilisation systems and imaging methods, aiming to improve the accuracy of treatment delivery. The shifts reported are almost all for IGRT sessions where the time interval between positioning the patient and starting treatment delivery is significantly shorter than in much longer adaptive MR-linac sessions [3, 12]; so they may underestimate the shifts that can occur for MRgART on an MR-linac.

The dosimetric differences reported are specific for the two example cases presented and so can only be indicative, as they depend on the size of the tumour, the volume of the target covered by high density parts of the ppd, the movement of the patient inside the mask, the entry points of the beams, etc. In addition, the longitudinal positioning of the patient on the ppd is set by the fixed position of the headrest integrated into the support. Therefore, depending on the patient height and conformation and the tumour position and size, the treatment fields may be differently located for each patient with respect to the ppd high-density areas.

The quantitative evaluation for only two rectum cancer patients presented in the study should not be viewed as a limiting factor, but as an opportunity to verify in the only two available clinical cases the effect of using such a system and as a means to alert other users to the issue. The results obtained for these two patients cannot be representative of a population, which makes this problem even more critical because there is no systematic way of taking this error into account. Effects must be assessed by each centre and over real-world treatment courses.

The use of thermoplastic masks for the treatment of pelvic areas is only one possible immobilisation systems ; alternative standard accepted systems used worldwide include supports for feet, knees and head, or vacuum cushions [30, 31]. However, thermoplastic masks are widely used, including significantly in China for pelvic treatments [29, 32]. The current Chinese National Guidelines for rectal radiotherapy [8] suggest thermoplastic masks for immobilization, as does Chinese expert consensus on clinical operational guidelines for CT simulation [7]. Therefore, it is important to carefully investigate the use of such devices in the treatment plan and in the Unity MR-linac system adaptive workflow. To retain the ppd and mask system, a possible solution could be to consider a correction approach, using MRI-visible markers on the ppd, to outline the position/s of the high-density ppd components on the scans, thereby directly providing their position relative to the patient’s inner anatomy. Another possibility is to avoid beam entry angles from the posterior, toavoid the high-density structures. However, plan optimisation without these beam entry options appears not to be a feasible solution for high-modulation plans, with acceptable plan optimization and delivery times, and with existing limitations regarding entry beam angles [33]. A further solution, as adopted in our department, is to use different immobilisation methods.

Improvements in the accuracy of treatment delivery are continuously sought. For patient setup, gains of 1–2 mm on different immobilization systems [17, 34] are welcome; moreover, in the daily routine, shifts or deviations larger than 1 mm and visible with IGRT can be realigned in each individual session. In complex MR-only adaptive workflows with the goal of high-quality adaptive radiotherapy delivery, it remains fundamental to limit as far as possible all sources of errors that can impact the final treatment, particularly where other compromises have to be accepted, such as rigid registration and ED assignment for dose calculation on the sCT [1].

The issue has been reported to Elekta as provider of the Unity system, with the response that importing couch structures separately into Monaco will be considered for inclusion in future releases. However, the problem remains for users with current versions of Monaco and ppd boards already currently supplied. This report is to draw attention to the problem to alert other centres to consider the issue and to modify methods to deal with it.

## Conclusion

In an adaptive workflow on the Unity MR-linac system, the use of a ppd with high-density components can introduce specific errors in the plan dosimetry if there are movements of the patient relative to the ppd board and the high-density structures are in (or close to) the treatment fields, due to the ppd not being visible on MR images and the way that the ppd is dealt with in current versions of the Monaco TPS. Considering the expectation of high-precision treatment, it is recommended not to use such a device on the MR-linac for such treatments, without modification to the device and the workflow, followed by careful clinical evaluation, or alternatively to use other immobilisation methods.

## Data Availability

The datasets generated during and analyzed during the present study are not publicly available due to participant privacy but are available from the corresponding authors upon request.
